# ATP Changes the Fluorescence Lifetime of Cyan Fluorescent Protein via an Interaction with His148

**DOI:** 10.1371/journal.pone.0013862

**Published:** 2010-11-05

**Authors:** Jan Willem Borst, Marieke Willemse, Rik Slijkhuis, Gerard van der Krogt, Sergey P. Laptenok, Kees Jalink, Be Wieringa, Jack A. M. Fransen

**Affiliations:** 1 Laboratory of Biochemistry, Microspectroscopy Centre, Wageningen University, Wageningen, The Netherlands; 2 Department of Cell Biology, Nijmegen Centre for Molecular Life Sciences, Radboud University Nijmegen Medical Centre, Nijmegen, The Netherlands; 3 Division of Cell Biology, The Netherlands Cancer Institute, Amsterdam, The Netherlands; University of California, United States of America

## Abstract

Recently, we described that ATP induces changes in YFP/CFP fluorescence intensities of Fluorescence Resonance Energy Transfer (FRET) sensors based on CFP-YFP. To get insight into this phenomenon, we employed fluorescence lifetime spectroscopy to analyze the influence of ATP on these fluorescent proteins in more detail. Using different donor and acceptor pairs we found that ATP only affected the CFP-YFP based versions. Subsequent analysis of purified monomers of the used proteins showed that ATP has a direct effect on the fluorescence lifetime properties of CFP. Since the fluorescence lifetime analysis of CFP is rather complicated by the existence of different lifetimes, we tested a variant of CFP, i.e. Cerulean, as a monomer and in our FRET constructs. Surprisingly, this CFP variant shows no ATP concentration dependent changes in the fluorescence lifetime. The most important difference between CFP and Cerulean is a histidine residue at position 148. Indeed, changing this histidine in CFP into an aspartic acid results in identical fluorescence properties as observed for the Cerulean fluorescent based FRET sensor. We therefore conclude that the changes in fluorescence lifetime of CFP are affected specifically by possible electrostatic interactions of the negative charge of ATP with the positively charged histidine at position 148. Clearly, further physicochemical characterization is needed to explain the sensitivity of CFP fluorescence properties to changes in environmental (i.e. ATP concentrations) conditions.

## Introduction

In a recent paper [1] we described that ATP induces changes in YFP/CFP fluorescence intensities in YFP/CFP based Fluorescence Resonance Energy Transfer (FRET) sensors. Although we presented this finding as a cautionary note, we also pointed out that this ability can possibly serve as basis for development of a new range of genetically encoded biosensors for monitoring ATP concentrations. ATP is the primary energy source in every living cell and knowledge about its temporal and spatial behavior is of great importance. Based on early observations [2], [3], [4] and recent own work [5], [6] a model of compartmentalized production and consumption of ATP is now emerging. Therefore, tools to monitor dynamic changes in the intracellular distribution of ATP at near physiological concentrations in living cells are now urgently needed. Recently, the development of Perceval, a new reporter for ATP:ADP ratios in cells was described [7]. Perceval is a circular permuted GFP variant, which upon binding of ATP to a domain derived from bacterial regulatory protein GlnK1, changes its fluorescence intensity. As the authors of this work point out, this sensor has its limitations and a ratiometric sensor based on FRET would still be welcome.

In our previous work we postulated that the ATP effect on CFP-YFP based FRET sensors occurred most likely via a direct quenching of the energy transfer step, possibly coupled to energy-induced charge displacement in the phosphate groups. We have applied fluorescence lifetime spectroscopy, which is generally accepted as one of the most reliable quantitative tools for this type of studies [8], [9], to get more insight into the physical basis and sequence of events involved. In this paper, we employed fluorescence lifetime spectroscopy to analyze effects of ATP on different fluorescent proteins in more detail.

## Methods

### Expression constructs and purification of proteins

For the bacterial expression of proteins the open reading frames of eCFP (CFP), Cerulean (CrFP), eYFP (YFP) and our previously described CFP-xa-YFP control construct [1], in which a Xa protease sensitive cleavage site is placed between the CFP-YFP chromophores, were amplified by a polymerase chain reaction (PCR) from the full-length cDNAs and appropriate segments were cloned into the pTYB11 vector (New England Biolabs, Impact vector system). The purification of the protein was performed as described in the manual of New England Biolabs, Impact vector system. The protein solutions were kept in 50 mM Hepes (pH 7.5) and stored in aliquots in −80 degrees.

For experiments in mammalian cells we used the same 6xHis-Tag containing vector designated CFP-xa-YFP as described before [1]. In addition a version in which the CFP part was exchanged with Cerulean, designated as CrFP-xa-YFP, was used.

For purification of the mammalian expressed proteins we used the Ni-NTA Qiagen kit (Qiagen, Hilden, Germany) according to the manufacturer's instructions.

### FRET measurements

COS-1 (ATCC CRL-1650) cells expressing CFP-xa-YFP or CrFP-xa-YFP proteins were lysed in a buffer containing 50 mM NaH_2_PO_4_, 300 mM NaCl, 10 mM Imidazole, 0,05% Tween 20, pH 8,0, by repeated freeze/thawing. Steady-state spectral fluorescence emission recordings of cleared extracts or purified protein in a Tris/NaCl buffer of pH 7.4 were obtained using a Shimadzu RF-5301 spectrofluorimeter (Shimadzu Corporation, Kyoto, Japan) using an excitation wavelength of 425 nm and a bandwidth of 10 nm.

### Fluorescence Lifetime Spectroscopy

Time-resolved fluorescence measurements were carried out using a mode-locked continuous wave laser for excitation and time-correlated single photon counting (TCSPC) as detection technique as described previously [10]. The samples were prepared either in Tris/NaCl buffer, pH 7.4 or in 50 mM Hepes, pH 7.5. ATP or MgATP were prepared in same buffer before addition to protein samples. The samples were excited with plane polarized light pulses (0.2 ps FWHM) at an excitation frequency of 3.8 MHz and both parallel- and perpendicular-polarized fluorescence intensities were detected. At 430-nm excitation, CFP fluorescence was detected with a 480.5-nm interference filter (Schott, Mainz, Germany; half-bandwidth of 5.4 nm). The sensitized emission of YFP fluorescence was detected with an OG 530 cut-off filter (Schott) and 557.6-nm interference filter (Schott; half-bandwidth 5.9 nm). The dynamic instrumental response function of the setup (40 ps FWHM) was obtained at the CFP or YFP emission wavelengths by using a solution of xanthione in ethanol as reference compound having an ultrashort fluorescence lifetime of 14 ps [11], [12]. The use of the reference convolution method [11] together with the current instrumentation enables determining fluorescence lifetimes with high accuracy and picosecond precision. Data analysis was performed using a model of discrete exponential terms. Global analysis of the experimental data was performed using the TRFA Data Processing Package of the Scientific Software Technologies Center (Belarusian State University, Minsk, Belarus) [13].

## Results

**Figure 1:**
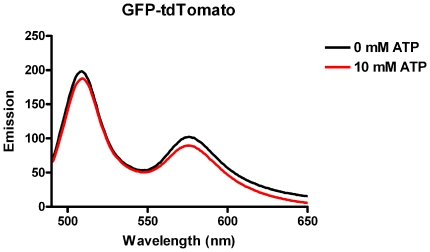
Effect of ATP on a GFP based FRET construct. Steady state fluorescence emission spectrum of a GFP-tdTomato construct ([15]) in the presence or absence of 10 mM ATP. Excitation of the GFP was at 480 nm excitation.

Previously, we demonstrated that the effect of ATP on FRET signals appeared to be independent of the linker sequence between the CFP and YFP moieties in all biosensors studied [1]. Although we did observe a decrease in energy transfer with increasing linker lengths (Data not shown, see also previous work [14]) no effect of the linker length was observed on the ATP-induced changes in YFP/CFP ratio. Interestingly, when we subsequently analyzed a range of constructs encoding sensors composed of different donor and acceptor fluorescent proteins [15] we found that the effect of ATP on the fluorescence intensity ratio's was only observed in constructs composed of CFP-YFP based versions. In Figure 1 the effects of ATP on a GFP-tdTomato [15] construct is shown as an example.

**Figure 2:**
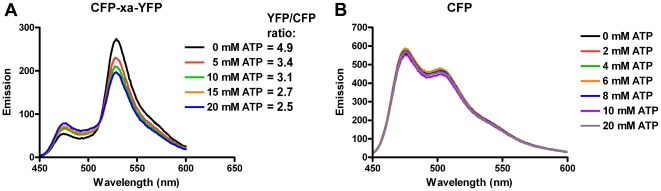
Effect of ATP on a CFP based FRET construct. Steady state fluorescence emission spectra of CFP-xa-YFP (A) and CFP alone (B) at different ATP concentrations. Excitation of the CFP was at 420 nm excitation.

To exclude the possibility that still other cellular factors, besides ATP, affect the FRET efficiency, we analyzed the effect ATP has on the FRET efficiency of bacterial expressed and purified CFP and YFP alone, and compared it with the effects it has on the dual-colored CFP-xa-YFP control construct. As can be seen in Figure 2A, the effects of ATP could be nicely reproduced for the CFP-xa-YFP construct but ATP did not influence the spectral properties of CFP (Fig. 2B) or YFP alone (data not shown).

**Figure 3:**
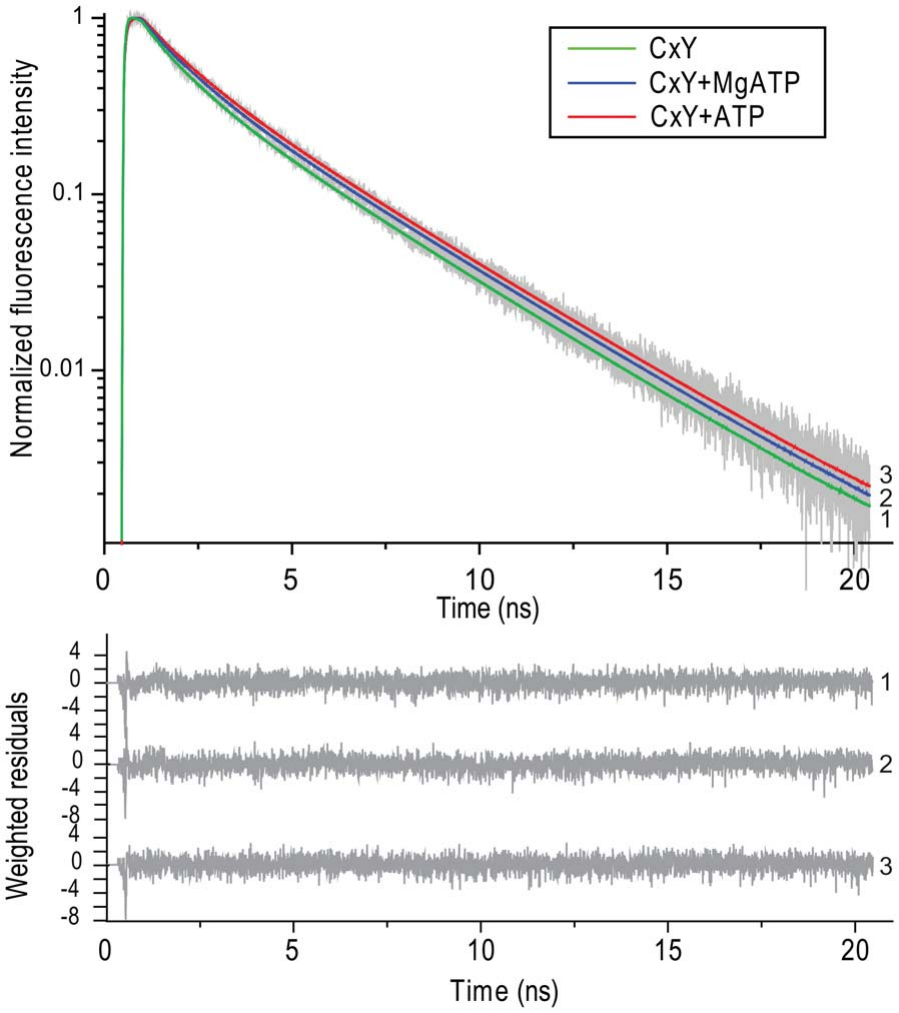
Fluorescence decay curves of CFP – YFP constructs. Normalized experimental (dotted line) and fitted (solid line) fluorescence decay curves of CFP-xa-YFP (curve 1), CFP-xa-YFP in the presence of 10 mM MgATP (curve 2) or 10 mM ATP (curve 3). The excitation wavelength was 430 nm and the detection wavelength of CFP emission was 480 nm. Weighted residuals are shown in the bottom panel and the recovered parameters (α, τ) are collected in Table 1.

**Figure 4:**
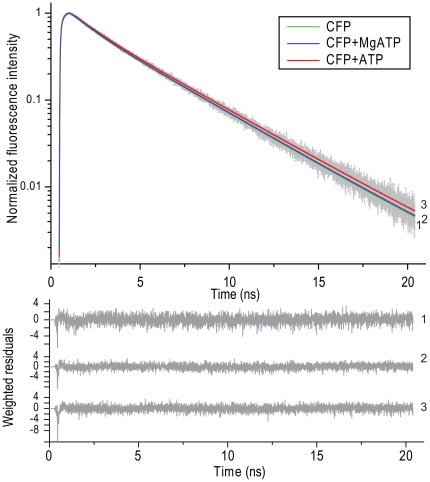
Fluorescence decay curves of purified monomeric CFP. Normalized experimental (dotted line) and fitted (solid line) fluorescence decay curves of CFP (curve 1), CFP in the presence of 10 mM MgATP (curve 2) or 10 mM ATP (curve 3). The excitation wavelength was 430 nm and the detection wavelength of CFP emission was 480 nm. Weighted residuals are shown in the bottom panel and the recovered parameters (α, τ) are collected in Table 2.

To answer the question whether the effect of ATP is the result of direct quenching of the energy transfer step or whether it is affecting the fluorescence characteristics of either one or both of the fluorescent moieties in the FRET sensor, we employed a more sensitive and quantitative method; time-resolved fluorescence spectroscopy [8]. Steady-state fluorescence spectroscopy of fluorescent sensors provides only information on average fluorescence properties of donor and acceptor molecules. The advantage of time-resolved fluorescence spectroscopy over steady-state fluorescence spectroscopy is that more detailed information on protein dynamics can be obtained. A fluorescent lifetime can be described as the average time a fluorescent molecule spends in the excited state. This is an intrinsic property of every fluorescent molecule, which is dependent on its environment but probe concentration independent. We have performed fluorescence lifetime measurements on individual fluorescent proteins; CFP, Cerulean (CrFP), YFP and the different CFP based sensors. Generally, in FRET constructs the fluorescence lifetime of the donor (in our case CFP or CrFP) is measured. Fluorescence lifetime measurements of the CFP-xa-YFP control construct showed a clear effect at increasing ATP concentrations on the fluorescence lifetime of the CFP (see table 1). The fluorescence decay curves of this sensor in the absence and presence of ATP and MgATP are depicted in figure 3. The calculated average fluorescence lifetime changed from 1.37 ns to 1.83 ns upon addition of 10 mM ATP. Fluorescence lifetime measurements of the purified samples of CFP and YFP alone showed no effect on the fluorescence decay kinetics of YFP (Table 2) but, interestingly, we observed an increase of the fluorescence lifetime of CFP. As shown in table 2 and figure 4, the fluorescence lifetime of CFP changes from 2.86 ns to 3.04 ns at 10 mM ATP. An increase of the fluorescence lifetime was already evident at 2 mM ATP. The data of monomeric CFP were analyzed using a bi-exponential decay model; the data of the CFP-xa-YFP and CrFP-xa-YFP constructs were analyzed using a 3-component decay model [16], [17]. Since previous experiments were performed by addition of MgATP, we tested also different concentrations of MgSO4. Interestingly, we again found a quenched fluorescence lifetime of CFP. The fluorescence lifetime of CFP decreased to almost the original value (2.90 ns). All the fluorescence lifetimes and the effects of ATP and MgATP are summarized in table 1 and 2.

**Table 1:** Fluorescence decay parameters of CFP – YFP constructs.

	*α_1_*	*τ_1_* (ns)	*α_2_*	*τ_2_* (ns)	*α_2_*	*τ_3_* (ns)	*<τ>* (ns)
CxY	0.37	0.18 (0.15–0.20)	0.31	1.05 (0.97–1.12)	0.31	3.17 (3.12–3.20)	1.37±0.04
CxY+ATP	0.32	0.24 (0.19–0.29)	0.33	1.19 (1.08–1.32)	0.42	3.25 (3.20–3.29)	1.83±0.07
CrxY	0.21	0.30 (0.23–0.39)	0.38	1.30 (1.18–1.45)	0.41	3.22 (3.17–3.28)	1.88±0.08
CrxY+ATP	0.19	0.31 (0.22–0.41)	0.37	1.34 (1.20–1.51)	0.44	3.24 (3.19–3.30)	1.98±0.09
CxY-H148D	0.31	0.39 (0.35–0.43)	0.33	1.60 (1.46–1.74)	0.36	3.53 (3.47–3.59)	1.92±0.07
CxY-H148D +ATP	0.29	0.53 (0.47–0.59)	0.35	1.89 (1.68–2.14)	0.36	3.70 (3.57–3.79)	2.15±0.13
CxY+MgATP	0.32	0.18 (0.14–0.21)	0.33	1.08 (0.99–1.17)	0.35	3.22 (3.17–3.26)	1.54±0.05
CrxY+MgATP	0.20	0.33 (0.24–0.43)	0.37	1.39 (1.24–1.58)	0.43	3.29 (3.22–3.36)	1.99±0.10

Fluorescence decay parameters of the CFP-xa-YFP (CxY), CrFP-xa-YFP (CrxY) and CxY-H148D in absence and presence of ATP or ATP where Mg (MgATP) is added.

*Note.* Values in parentheses are the 67% confidence limits. The average fluorescence lifetime (in ns) is calculated as <*τ>*  =  *α*_1_ *τ*_1_ + *α*_2_ *τ*_2_ + *α*_3_ *τ*_3_.

**Table 2:** Fluorescence decay parameters of purified monomeric proteins.

	*α_1_*	*τ_1_* (ns)	*α_2_*	*τ_2_* (ns)	<τ> (ns)
CFP	0.28	1.03 (0.95–1.09)	0.72	3.57 (3.56–3.59)	2.86±0.03
CFP+ATP	0.26	1.23 (1.13–1.32)	0.74	3.68 (3.64–3.7)	3.04±0.04
CFP+MgATP	0.28	1.11 (1.02–1.18)	0.72	3.60 (3.56–3.62)	2.90±0.05
CrFP	0.33	1.80 (1.66–1.93)	0.67	3.75 (3.69–3.82)	3.10±0.08
CrFP+ATP	0.32	1.76 (1.62–1.89)	0.68	3.74 (3.69–3.80)	3.10±0.07
CrFP+MgATP	0.31	1.70 (1.56–1.82)	0.69	3.71 (3.66–3.76)	3.09±0.07
YFP					3.05±0.02
YFP + ATP					3.04±0.02
YFP+MgATP					3.06±0.03

Fluorescence decay parameters of the CFP, CrFP and YFP in absence and presence of ATP or MgATP.

*Note.* Values in parentheses are the 67% confidence limits. The average fluorescence lifetime (in ns) is calculated as <*τ*> =  *α*_1_ *τ*_1_ + *α*_2_ *τ*_2_.

**Figure 5:**
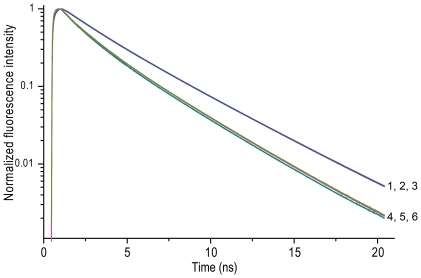
Fluorescence decay curves of purified CrFP and CrFP-xa-YFP. Normalized experimental (dotted line) and fitted (solid line) fluorescence decay curves of CrFP (curve 1; no ATP, curve 2; 10 mM MgATP, curve 3; 10 mM ATP), and CrFP-xa-YFP (curve 4; no ATP, curve 5; 10 mM MgATP, curve 6; 10 mM ATP). The excitation wavelength was 430 nm and the detection wavelength of CFP emission was 480 nm. The recovered parameters (α, τ) are collected in Table 1 and 2.

The fluorescence lifetime properties of CFP are rather complicated due to the existence of two different conformations exhibiting two different lifetimes [18], [19]. For this reason a CFP variant [20], Cerulean (CrFP), was generated exhibiting mono-exponentially decay. The main difference of this variant compared to CFP is the change of histidine148 into an aspartic acid. Surprisingly, analysis of purified monomeric Cerulean showed no effect on the fluorescence lifetime at increasing amounts of ATP. In figure 5 the fluorescence decay curves are shown and the calculated fluorescence lifetimes are summarized in table 2.

**Figure 6:**
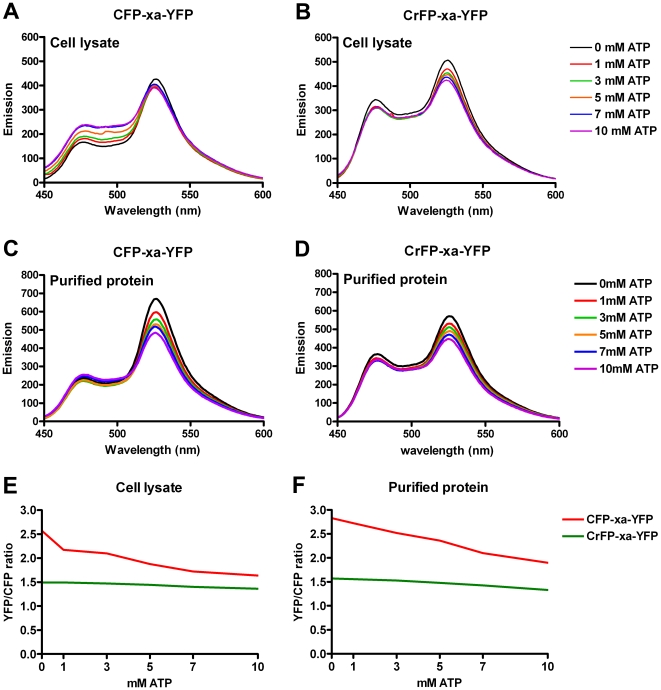
Fluorescence emission spectra of proteins expressed in Cos-1 cells. Steady state fluorescence emission spectra of Cos-1 cells expressing CFP-xa-YFP (A, C) and CrFP-xa-YFP (B, D) in cell lysates (A, B) and of Ni-NTA purified protein (C, D) at different ATP concentrations. Under both conditions the YFP/CFP ratio shows a clear ATP dependent change in the CFP-xa-YFP construct whereas hardly any change was observed in the CrFP-xa-YFP construct (E, F). Excitation of the CFP was at 425 nm excitation.

Next, we assessed Cerulean's properties in the context of FRET in mammalian cells, by changing CFP for Cerulean in our CFP-xa-YFP constructs. Again, we compared directly the effects of increasing amounts of ATP on the CFP and Cerulean-based constructs by spectral analysis in cell lysates (Fig. 6A, C and E) and on the purified monomeric proteins (Fig. 6B, D and F). In the spectral analysis, the effect of ATP on YFP/CFP ratios (Fig. 6E and F and table 3) was again only observed in the construct containing CFP and absent in the CrFP containing construct. Also, fluorescence lifetime measurements showed changes on donor lifetimes only in constructs containing CFP moieties (Fig. 5 and table 2). Finally, by specifically mutating the histidine at position 148 into an aspartic acid in the CFP-xa-YFP construct we could reproduce the lifetime results obtained by the CrFP-xa-YFP construct (See table 1).

**Table 3:** YFP/CFP peak ratios of CFP – YFP constructs.

mM ATP	0	1	3	5	7	10
	Lysate:					
CFP-xa-YFP	2.57	2.17	2.10	1.88	1.72	1.64
CrFP-xa-YFP	1.49	1.49	1.47	1.44	1.40	1.36
	Purified Protein:					
CFP-xa-YFP	2.83	2.72	2.52	2.36	2.10	1.90
CrFP-xa-YFP	1.57	1.56	1.53	1.48	1.43	1.43

YFP/CFP peak ratios in Cos-1 cells expressing CFP-xa-YFP and CrFP-xa-YFP in cell lysates and in Ni-NTA purified protein from these lysates (see Materials and Methods) at different ATP concentrations.

Taken together, our observations lead us to conclude that the effect of ATP on the fluorescence characteristics of CFP occurs specifically via possible electrostatic interactions of the negative charge of ATP to the positively charged histidine residue at position 148 within the CFP molecule.

## Discussion

Previously, we postulated that ATP could have an effect on FRET signals in CFP-YFP based sensors by quenching of the energy transfer between the two fluorophores. Here, we show by time-resolved fluorescence spectroscopy that the effect of ATP is not via quenching of the energy transfer step but via a direct interaction of ATP with the histidine residue at position 148 of CFP. The advantage of fluorescence lifetime measurements over steady-state fluorescence is that fluorescence lifetimes are concentration independent but can be influenced by the local environment of the fluorophore and can therefore be used to map this environment (pH etc.). It is known that the pH can change the protonation of the chromophore, thereby affecting the absorption characteristics and consequently the fluorescence lifetime of visible fluorescent proteins [21]. In our experiments we can exclude pH effects as most samples were prepared with and without ATP/MgSO4 at identical pH-values.

It is known that the presence of multiple fluorescence lifetimes in CFP is due to conformational adaptation, where two different conformations of CFP result from the alternate displacement of two hydrophobic residues (Tyr145 and His148) [18], [19] to the surface of the protein. Mutation of His148 to aspartic acid results in an almost completely single exponential fluorescence decay, a feature that was rationally engineered in Cerulean [20]. The recent described X-ray structure of Cerulean [22] now provides a structural explanation for the described change in the fluorescence decay compared to the CFP variant. It has been shown recently that the fluorescence decay of Cerulean is also not exactly monoexponential [23]. As shown here, the His148 residue in CFP is involved in binding ATP, thereby effecting the fluorescence lifetime properties upon ATP application.

In the two conformations of the original CFP, the side chain of His148 is either oriented towards the chromophore or positioned at the outside of the protein. Since the effect of ATP is seen on all lifetime components of CFP, we now speculate that ATP dynamically associates with this residue via π-π or cation-π interactions [24], [25] or indirectly by influencing the conformation of the side chain. However, a direct electrostatic interaction with the negatively charged ATP molecule is also possible since the pKa of the side chain of histidine is about 6 [26]. Our experiments have been performed at pH 7.5, which results in a partially positive charge of the histidine residue. The fluorescence lifetimes of purified monomeric CFP were calculated using a two-component fit model. A closer look at the data showed that it is the long component that is mainly affected upon ATP addition. In principle two effects are mixed during these analyses. First, the minor or major conformation of CFP determines the binding of ATP to the histidine. Secondly, at lower pH the side chains of histidine becomes more positively charged. Using lower pH has a direct influence on the chromophore of CFP causing a change in fluorescence lifetime properties. A third explanation may be the stacking of ATP to the histidine thereby changing the local conformation of CFP. As a result, flipping of the side chain can open up a pore, thereby giving access for quenching molecules to the chromophore (see [19]).

Either way, since His148 is one of the amino acid residues closest to CFP's chromophore, we can easily explain that changing the conformation of CFP or charge of the chromophore can have an effect on the CFP fluorescence properties. These effects become even more drastic in CFP/YFP FRET biosensors because distance and/or orientation may affect the fluorescence characteristics and thereby change the FRET readout of CFP/YFP based biosensors.

During the course of our studies, Imamura et al. [27] published a FRET based sensor for the visualization of ATP levels inside single living cells, which is also based on CFP and YFP but contains the epsilon subunit of the bacterial F(o)F(1)-ATP synthase as ATP sensing domain. However, in their control studies they do not see a direct effect of ATP on their sensor. We can explain this observation by the fact that these authors study changes in FRET ratios using micromolar concentrations of ATP, while we use millimolar ATP concentrations. We therefore conclude that the sensitivity of their ATP binding linker for ATP is much higher than an electrostatic interaction of ATP with the histidine at position 148, making their sensor better suited for the analysis of the intracellular distribution of ATP concentrations in living cells. In cells or conditions with large and sudden fluctuations in local or global ATP concentration however, we still conclude that findings should be interpreted with caution if genetically encoded CFP-YFP based biosensors are used.

Finally, combining recent spectroscopic work of Villoing et al. [28], showing that both CFP and its H148D mutant exhibit highly complex temperature and pH dependent fluorescence decays, and our work presented here, illustrates that we still have only limited understanding of the complex and heterogeneous fluorescence properties of CFP and the H148D variant Cerulean or the effects thereon of small molecules in its environment. Therefore, further physicochemical characterization is needed to explain the sensitivity of CFP fluorescence to changes in solute conditions, including changes in ATP-Mg concentration. Ultimately, better understanding of the emissive properties of fluorescent proteins may lead to a more rational design of FRET sensors.
